# The Impact of a Yoga-Based Mindfulness Intervention versus Psycho-Educational Session for Older Adults with Mild Cognitive Impairment: The Protocol of a Randomized Controlled Trial

**DOI:** 10.3390/ijerph192215374

**Published:** 2022-11-21

**Authors:** Maryam Farhang, Graciela Rojas, Pablo Martínez, Maria Isabel Behrens, Álvaro I. Langer, Marcela Diaz, Claudia Miranda-Castillo

**Affiliations:** 1 Escuela de Enfermería, Facultad de Salud y Ciencias Sociales, Universidad de Las Américas, Santiago 8370040, Chile; 2 Millennium Institute for Research in Depression and Personality (MIDAP), Santiago 8380453, Chile; 3 Millennium Institute for Care Research (MICARE), Santiago 8370134, Chile; 4 Departamento de Psiquiatría y Salud Mental, Hospital Clínico Universidad de Chile, Santiago 8380456, Chile; 5 Millennium Nucleus to Improve the Mental Health of Adolescents and Youths, Imhay, Santiago 8380455, Chile; 6 Faculté de Médecine et des Sciences de la Santé, Université de Sherbrooke, 150, Place Charles-Le Moyne, Longueuil, QC J4K A08, Canada; 7 Centro de Investigación Clínica Avanzada (CICA), Hospital Clínico Universidad de Chile, Santiago 8380456, Chile; 8 Departamento de Neurología y Neurocirugía, Hospital Clínico Universidad de Chile, Santiago 8380456, Chile; 9 Departamento de Neurociencia, Facultad de Medicina, Universidad de Chile, Santiago 7800284, Chile; 10 Departamento de Neurología y Psiquiatría, Clínica Alemana Santiago, Universidad del Desarrollo, Santiago 7800284, Chile; 11 Mind-Body Lab, Institute of Psychological Studies, Austral University, Valdivia 5110566, Chile; 12 Clínica Psiquiátrica Universitaria, Hospital Clínico, Universidad de Chile, Santiago 7800284, Chile; 13 Faculty of Nursing, Universidad Andres Bello, Santiago 7550000, Chile

**Keywords:** older people, mild cognitive impairment, yoga, mindfulness, mind-body intervention

## Abstract

Background: There is a global agreement in the medical community that a significant proportion of dementia cases could be prevented or postponed. One of the factors behind this agreement comes from scientific evidence showing that mind-body interventions such as mindfulness and yoga for the elderly have been related to a range of positive outcomes, including improved cognition performance in seniors with mild cognitive impairment (MCI). Objective: This study aims to evaluate the effectiveness of a yoga-based mindfulness intervention (YBM) versus psychoeducational sessions for older adults with MCI attending Hospital Clinic Universidad de Chile in Santiago. Method: Two-arm, individually randomized controlled trial (RCT) will be carried out at Clinical Hospital Universidad de Chile in Santiago. Older people over 60 years with any type of MCI using a score < 21 in the Montreal Cognitive Assessment (MoCA) test and a score of 0.05 in the Clinical Dementia Rating (CDR) Scale; and with preserved activities of daily living will be randomly assigned with an allocation ratio of 1:1 in either the yoga-based mindfulness intervention or the active control group based on the psycho-educational program. People who have performed yoga and/or mindfulness in the last 6 months or/and people with a psychiatric clinical diagnosis will be excluded from the study. Montreal Cognitive Assessment, the Lawton Instrumental Activities of Daily Living Scale (IADL), the Barthel Index (BI), the Pemberton happiness index, the Geriatric Anxiety Inventory (GAI) as well as the Geriatric Depression Scale (GDS-5) will be administered by blinded outcomes assessors before random assignment (Pre-test), the week following the last session of the intervention (post-test), and then after 3- and 6-months follow-up. Results: The YBM intervention protocol based on a video recording has been adapted and designed. This is the first RCT to examine the effects of a yoga-based mindfulness intervention in improving cognitive and physical functions and mental health outcomes for Chilean elderly diagnosed with MCI. It is expected to be implemented as an acceptable and effective non-pharmacological option for older people with MCI. Conclusion: Providing evidence-based programs such as preventive therapy for Alzheimer’s disease has relevant implications for public mental health services in Chile.

## 1. Background

Demographers and social scientists have noted that a transition toward an older global population has been underway in many parts of the world. In October 2022, new projections from the World Health Organization (WHO) revealed that by 2050 the proportion of the global population who are over 60 years old would increase from 12% to 22% [1]. By that time, the proportion of people over 65 years old will rise to 16 percent of the global population, compared to 10 percent in 2022 [2], which confirms the demographic transition toward a more elderly global population [3]. This has not only been occurring in developed countries such as Japan, The European Union, and The United States, but it has also been occurring in developing countries as well. By the year 2090, the Latin American and the Caribbean region is expected to have the highest percentage of people aged over 60, at around 36 percent of the total regional population [3]. It is estimated that the number of Chileans over 60 years old will increase from the current 15.7% of the population to 32.9% by 2050 [4], and the proportion of people older than 80 will reach 10.3% [5].

Along with this shift towards a more aged population, there has been a greater incidence of illnesses and disorders that are more commonly associated with this group. One of these is dementia, a progressive decline of the cognitive ability which predominantly affects people over the age of 60. It has emerged as a main public health concern [6]. It is projected that the number of people in the world with dementia will reach 131.5 million by 2050 [7]. This disorder has become more prevalent in every country where the elderly population has increased. Chile is one of these countries where dementia has become a significant cause of disability among the elderly, with a prevalence of 10.4% (10.6% women, men 10.1%) [8]. The need for preventive treatment is particularly evident if we consider that WHO estimates that there will be a 77% increase in the cases of dementia by 2030 in Chile, Argentina, and Uruguay [9].

Dementia is usually preceded by a phase called mild cognitive impairment (MCI), a stage between the normal cognitive changes caused by aging and early dementia [10]. It encompasses a wide range of cognitive impairments such as memory, executive function, attention, language, or visuospatial skills [11]. Ample evidence has emerged that MCI has become equally prevalent among this segment of the population. A 2016–2017 National Health Survey reported that 10% of adults over 60 years old had suspected MCI, reaching 21% in those over 80 [12]. In the global sample, the prevalence of MCI for ages 60 to 64 years old was 6.7% [13]. However, in adults over 65 years, the prevalence increases to 14.8% and reaches 25.2% in those over 80 [13]. A number of studies have provided evidence that people with MCI are at greater risk of developing Alzheimer’s disease (AD) [14]; 5–15% of people with MCI are diagnosed with AD each year [15].

In addition, the elderly often experience depression and anxiety, which are common in people with MCI [16]. Recent findings indicate that people with MCI have a greater risk of developing the depressive disorder and anxiety disorder [17]. Previous studies show that the proportion of people with MCI who have depressive symptoms ranges between 36% to 63% [18]. Anxiety symptoms are equally frequent, with rates between 10 and 74% [16]. Chileans over 65 diagnosed for the first time with depression presented a greater link with cognitive impairment [19]. Other studies have also shown that depression and anxiety are risk factors for MCI [10,20] and that these conditions often precede the development of AD [21].

The decline in cognitive ability causes difficulty for older people in executing their daily activities [22] and has been associated with declines in functional status, quality of life, and increased dependency as well as mortality [23]. Albala [24] points out that the loss of physiological functioning because of aging reduces the resistance to stressors, leading to the greater vulnerability of older adults and to the development of frailty syndrome [24]. Frailty is strongly associated with cognitive impairment and depression in older Chilean adults [24].

Physical and mental health have been found to be related to subjective well-being. Many older adults experience lower levels of well-being due to such decline in their physical and cognitive functions [25] as well as social losses and chronic conditions, limiting their activities and leading to depression and anxiety [26]. Therefore, new interventions are needed to address multiple aspects of well-being in the elderly, helping them to maintain good health and to lead independent lives.

These data and facts highlight the importance of offering adequate care to the elderly population through treatments that bring about improvements in their cognitive and physical function. However, after numerous clinical trials, there are still no effective drugs approved by the Food and Drug Administration (FDA) for the treatment of MCI [27] or for postponing the progression to dementia [28].

Therefore, non-pharmacological therapies based on mind-body exercises, such as yoga and mindfulness, have been found to be feasible and effective intervention options for older adults with MCI [29,30,31,32].

Yoga is an ancient mind-body practice rooted in India whose main emphasis is to arrive at the “here and now” by using physical postures (asanas), breath control (pranayama), and meditation (dyana) [33]. Yoga has been known to boost physical and psychological well-being [34,35,36,37,38], reduce depression and anxiety [38], as well as to delay frailty in older adults [39]. Yoga-related interventions for the elderly are safe, practical, and acceptable practices for the strengthening of cognitive functions of those suffering age-related cognitive deterioration [40]. Findings of a study conducted by Tremont et al. [41] point out that the yoga intervention was safe, feasible, and effective for elderly patients with MCI. The authors obtained preliminary proof that yoga can ameliorate visuospatial functioning in MCI patients [41]. The results of the same study considered a reduction in stress as a potential mechanism for the yoga intervention [41]. Several major studies have demonstrated that cognitively impaired older adults could benefit from cognitive improvement through yoga intervention. In a study conducted by Chobe et al. [42], yoga intervention significantly improved global cognition as well as other cognitive abilities, including learning, attention, processing speed, and working memory among elderly persons with MCI.

Furthermore, an earlier study conducted by Gothe and McAuley [43] showed some beneficial effects of Hatha yoga on cognitive functions, including attention and processing speed, as well as executive function and memory [44]. In another study, the results showed that yoga, breathing exercises, and the practice of mindfulness meditation boosted brain wave activity as well as amygdala and frontal cortex activation in older people [45]. Moreover, yoga intervention demonstrated statistically significant development in the executive function of elderly patients with MCI as compared to memory enhancement training (MET) and a larger effect on depressive symptoms and resilience [30]. A previous study also confirmed that kundalini yoga is associated with improved neural activity and brain structural alterations associated with executive function [46], as well as improved memory performance, awareness, and attention in seniors with MCI [47].

Mindfulness therapy involves developing awareness of the present moment in a non-judgmental manner. A common type of intervention based on this therapy is called Mindfulness-based stress reduction (MBSR), which teaches mindfulness meditation and yoga [48]. MBSR may be categorized in terms of formal activities such as body scanning, breath meditation, loving-kindness meditation, and yoga and informal practices such as awareness in daily routines like mindful eating, walking, and communicating [49].

Due to its potential to reduce the main risk factors of dementia in late life, including depression, anxiety, and stress, accompanied by sleep disorders, mindfulness therapy has been found as a practical and efficacious intervention for strengthening the cognitive function of people with MCI and delaying the progress of MCI to AD [29,32,50]. Numerous studies indicate that such training can be an effective alternative treatment. Initial findings by Larouche et al. [51] showed that mindfulness-based intervention could prevent memory loss and can improve mood and quality of life in people with MCI. Findings of a recent pilot feasibility study in MCI patients indicated that MBSR is a potentially promising intervention with beneficial effects on cognition, psychological health, and the immune system [52]. In a study conducted by Fam et al. (2020), elderly with MCI who practiced mindfulness had better brain network efficiency and neuro-cognitive function rather than the control group. In this study, the author suggested that mindfulness may be of benefit to the older adult population with early stages of cognitive deterioration.

A further study conducted by Kinjal et al. [53] found that elderly with MCI receiving eight weeks of mindfulness-based training presented significant improvements in global cognition, with significant developments in delayed memory than participants in the cognitive rehabilitation therapy group. The results of a recent review indicated that such interventions improved cognitive function, motor control, and mindfulness, resulting in a reasonable reduction in the risk of falling, a decrease in symptoms of depression and stress, and a lower risk of dementia in adults with MCI [29]. A meta-analysis also suggests that mind-body training such as yoga and mindfulness positively impact attention, working memory, executive function, and global cognition [54]. However, inconsistent results of other meta-analyses conducted by Han [55] found no significant effect of mindfulness-based interventions on psychological outcomes, memory, and overall cognitive functions compared to comparison groups.

The results of a randomized, controlled pilot clinical trial showed that MCI participants in MBSR had increased functional connectivity within the default mode network (DMN), along with reduced hippocampal atrophy as compared to the usual care group [56]. Different findings suggest that mindfulness could arrest the cognitive decline in MCI as well as prevent the pathological symptoms of AD, namely progressive loss in the hippocampus and grey matter, along with decreased functional connectivity in the DMN [56,57,58].

The results of a study by Wong et al. [32] showed that cognitive function and mindfulness levels were enhanced after finishing a mindfulness intervention program. It also revealed that older adults with MCI who meditated for more than the average amount of time between program completion and one-year follow-up showed more significant gains in their cognitive and daily functioning [32].

Based on the evidence, applying an appropriate body-mind intervention for older people is effective in improving cognitive function, functional independence, and mental health outcomes, thereby delaying frailty and improving well-being in this population. However, comparatively few randomized, controlled trials (RCTs) interventions have been done, mainly in elderly patients with mild MCI, who are at increased risk of developing dementia. Furthermore, Chile’s epidemiological profile, characterized by the tendency towards a growing proportion of elderly adults with an increased incidence of dementia and mental illnesses, has prompted the search for an effective non-pharmacological intervention incorporating a mind-body component to improve cognitive and physical functions as well as mental health outcome. In addition, there have been no studies to demonstrate the effects of a yoga-based mindfulness intervention on cognitive decline in the elderly with MCI in Chile. Clearly, evidence from Chile and other countries in Latin America is needed. To address this, a study of early intervention using a yoga-based mindfulness component to prevent dementia in Chile is hereby proposed. Hence, a yoga-based mindfulness component would be utilized to design the intervention.

General Objective: To compare the effectiveness of yoga-based mindfulness (YBM) intervention versus psycho-educational sessions (PES) in improving cognitive and physical functions and mental health outcomes for older adults with mild cognitive impairment (MCI).

Specific Objectives:

(1) To compare the change in cognitive function scores between older adults with MCI receiving a YBM intervention versus those receiving a PES intervention.
(2) To compare the change in physical function scores between older adults with MCI receiving a YBM intervention versus those receiving a PES intervention.
(3) To compare the change in well-being scores between older adults with MCI receiving a YBM intervention versus those receiving a PES intervention.
(4) To compare the change in anxiety and depression scores between older adults with MCI receiving a YBM intervention versus those receiving a PES intervention.
(5) To assess the correlation between cognitive function and physical function in older adults with MCI, controlling for psychological variables (depression, anxiety, well-being), and group (YBM intervention or participating in PES)

**Hypothesis 1.**  *Older adults with MCI in the YBM intervention will have a different change in their cognitive function—as measured through MoCA assessment, before and after the intervention—than those in the PES intervention (with the same measures, before and after)*.

**Hypothesis 1.1.**  *Older adults with MCI in the YBM intervention will have a different change in their physical function—as measured through the Barthel Index (BI) and The Lawton Instrumental Activities of Daily Living Scale (IADL) before and after the intervention—than those in the PES intervention (with same measures, before and after)*.

**Hypothesis 1.2.**  *Older adults with MCI in the YBM intervention will have a different change in their well-being—as measured through the Pemberton happiness index, before and after the intervention—than those in the PES intervention (with the same measures, before and after)*.

**Hypothesis 1.3.**  *Older adults with MCI in the YBM intervention will have will have lower anxiety and depression levels—as measured through the Geriatric Anxiety Inventory (GAI) and the Geriatric Depression Scale (GDS-5), before and after the intervention—than those in the PES intervention (with same measures, before and after)*.

**Hypothesis 2.**  *Cognitive function will have an effect on physical function in older people with MCI, mediated by psychological factors (depression, anxiety, and well-being)*.

## 2. Method

### 2.1. Trial Design

**Figure 1:**
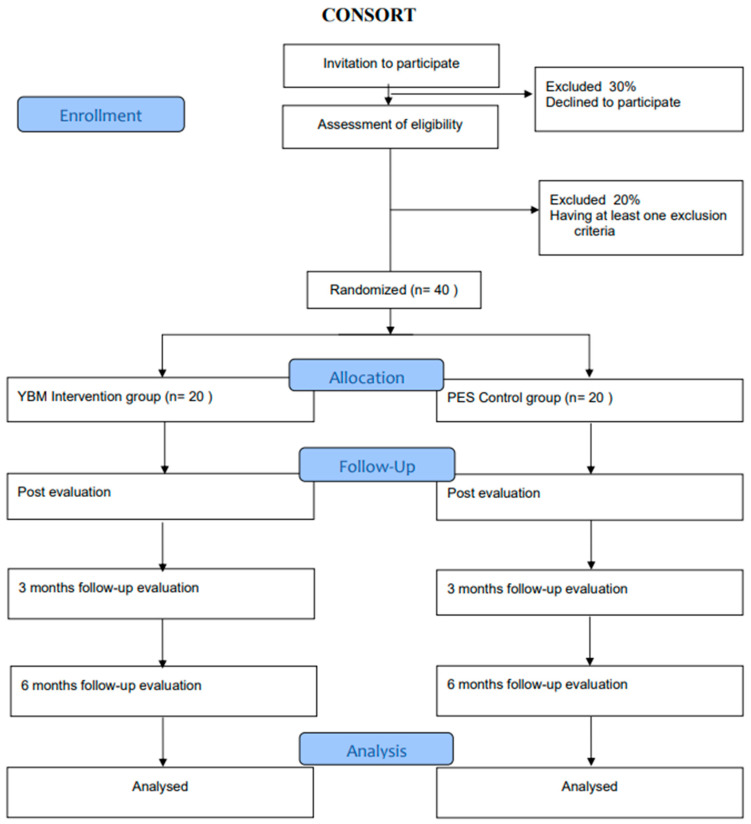
Participant flow through the study.

This is a two-arm, individually randomized controlled trial, superiority with an allocation ratio of 1:1, assessor-blinded, and 3- and 6-months follow-up. This study protocol has followed the SPIRIT guidelines [59]. Trial registration: A protocol for this study has been registered retrospectively at the ISRCTN registry on 28 April 2022. Identifier: ISRCTN13063260. https://www.isrctn.com/ISRCTN13063260, accessed on 28 April 2022. An overview is presented in Figure 1.

### 2.2. Study Setting

Participants will be recruited at Universidad de Chile Clinical Hospital in Santiago, Chile.

### 2.3. Participants

The target population corresponds to people aged 60 years and more with any type of MCI, and the eligibility criteria for the selection of the sample consisted of the following:

- Having 60 years and above,
- People who have MCI are diagnosed by three criteria:
  ➢ by a neurologist and/or psychiatrist, and/or geriatrician, and/or neuropsychologist. through evaluation by saying that they had memory problems confirmed by an informant (in case they have a formal or informal caregiver or simply, their partner), (but without problems in their activities of daily living);
  ➢ and with a score < 21 on the Montreal Cognitive Assessment (MoCA < 21).
- Without dementia, according to the criteria of the Clinical Dementia Rating Scale (CDR), a score of 0.05.

The Exclusion criteria would be:

- People who performed yoga and/or mindfulness within the last 6 months
- The presence of a psychiatric clinical diagnosis; or neurological/cerebrovascular condition.
- Presence of a disabling physical illness or/and presence of a disability that limits and/or impede communication, such as major impairments in eyesight, and/ hearing or upper limb motor movements, and/or other health problem that would interfere with regular yoga and mindfulness practice.

Attendants to the secondary care center (clinical hospital Universidad de Chile) who meet these criteria will be invited to participate in the study and will be randomized to an experiment or control group.

### 2.4. Recruitment and Informed Consent Process

Within the clinical hospital Universidad de Chile, users 60 years old and above that are receiving care at the clinic will be invited to participate in the study and will be referred by secondary care professionals (Physicians derived from health networks can be informed post-randomization). Prior to inclusion in the study, a psychiatrist, neurologist, or geriatrician will determine the patient’s capacity to consent to participate in the study before considering his/her inclusion. Therefore, qualified recruiters from the trial will contact potential participants (e.g., MoCA < 21) for evaluation of the initial eligibility criteria for the study, and before starting the evaluation process, overall characteristics of the trial will be explained to participants, and verbal informed consent will be obtained from them during the recruitment phone call. Participants who meet the inclusion criteria and agree to participate will be evaluated through a trained evaluator, with a battery of evaluation instruments using a telephone-based survey, and then they will be randomized to either the intervention or control group. Outcome assessments will also be conducted through these evaluators, blinded to the allocation of the intervention. However due to the nature of the psychosocial intervention, it will not be possible to blind the MCI participant. The secondary care team will be informed regarding MCI patients’ assignments after randomization.

To ensure a sufficient recruitment flow, recruiters will work on a daily basis in the morning under the supervision of the person in charge, who will be in close contact with the research team. To facilitate the work of the recruiters and to invite potential participants to contact them, leaflets and banners with basic information about the study will be available at the clinical hospital Universidad de Chile. Recruiters will also be in direct contact with neurologists or psychiatrists about each potential participant.

### 2.5. Randomization

The outpatient clinic within Clinical Hospital at Universidad de Chile will be allocated to the intervention or the comparator with a 1:1 ratio based on the random computer-generated numbers, using simple random sampling by sequentially numbered. In order to generate a random allocation sequence, a balanced block randomization will be used with a randomly varying block size of 4. A member of the research team who is not involved in the recruitment process in the outpatient clinic will oversee the generation of the random sequence and the allocation of the interventions. Allocation will be kept concealed from the recruiters by hiding the process from executive personnel of the Clinical Hospital, who will be obviously instructed not to contact health care staff at the participating centers. Randomization will be carried out once the initial evaluation has been carried out for all participants, avoiding the bias that could occur when randomization during the recruitment process.

### 2.6. Sample Size

The sample size is estimated based on changing the primary outcome (MoCA) according to the MCID/MCIC (Minimal Clinically Important Difference or Change), which has been established in a study performed by Wong et al. [60]. This Study suggested that a change of 2.0 can be considered as a reasonable minimum, clinically important difference for the main outcome variable (MoCA). Therefore, based on the previous study [32] examining mindfulness’s effect on the cognitive function of seniors with MCI, we estimated the effect size to be 0.5. Hence, we required 18 participants for each arm to have a power of 80% to detect statistical significance at a 5% level which was determined based on an analysis performed in the data of Wong et al. [32] using the Stata 17. Considering potentially a drop-out rate of 20%, 22 participants will be required to be allocated to each group. Therefore, the targeted total sample size will be 44 in order to sustain enough power in the present study.

## 3. Intervention Program

The Yoga based mindfulness intervention (YBM) will be based on video recording according to the following characteristics: (a) information about yoga and mindfulness will be delivered to seniors through specific exercises in each session (b) The mindful chair-based Hatha yoga will be used with an emphasis on proper Hatha yoga physical exercise or body awareness exercises [60,61] and mindfulness. If they find themselves unable to do a particular posture, they should be free to skip it and engage with their bodies with the attitude of kindness and acceptance, and after the completion of each posture, the participants will be asked to be mindful of muscles they will be tensing, stretching and which one will be used to keep their body balanced; (c) The mindfulness component will be based on (MBSR) guideline developed by Kabat-Zinn [48] which has been adapted from mindfulness protocol developed by Wong [32] for older people with MCI. The program will include both formal and informal practices. Participants will be taught to practice formal mindfulness techniques such as “body scan”, and “breath” meditations to observe and maintain attention on an object of meditation without judgment and reactivity even when one becomes distracted by drifting thoughts or unpleasant emotions and ‘loving-kindness’ meditation to cultivate kindness towards all beings, including oneself [32]. The informal practice involves spreading awareness to daily experiences such as mindful walking and eating [32]. The informal practice will also include mental exercises such as reading and solving puzzles, as well as being aware of daily interactions and other ADLs.

### 3.1. Procedures

Participants who would be assigned to the experimental group will receive the Yoga-based mindfulness intervention (YBM) for an hour, once weekly, for a total of 8 weeks. The YBM intervention program has been adapted to the needs, goals, and levels of understanding of MCI patients and will be executed based on a video that has been recorded by facilitators who have knowledge about yoga (M.F.) and mindfulness (M.D.) and who are different from those who will act as outcomes assessor and will be sent to the program attendees through WhatsApp application in order to watch and listen to on personal phones, computers or tablets. The control group will receive a passive psychoeducation with general information about wellness, self-care in health, and the promotion of exercise that will be delivered via text through the WhatsApp application each week.

### 3.2. Criteria for Stopping or Modifying Allocated Interventions

The neurologist who is in contact with patients for their treatment will be informed If patients allocated to the intervention or to the comparator present worsening of their physical or cognitive function at some point in the trial. Interventions will likewise be interrupted if a patient requests to withdraw from the study. Accordingly, this information will be informed to the affected participant and the Scientific Ethics Committee of the Clinical Hospital of the Universidad de Chile.

### 3.3. Plans to Improve Adherence to Intervention Protocols

Adherence to trial protocols will be ensured through participants’ satisfaction with the program. Therefore, they will be given telephone calls in order to keep a record of each session with the patients, and in case they have a doubt regarding the intervention program and contents. Moreover, the intervention program will be explained to their caregivers, and they will also be contacted in order to have their feedback about the intervention program.

### 3.4. Relevant Therapies and Interventions Allowedor Prohibited during the Trial

The patients would continue with their usual treatment, and that other interventions indicated by the doctor would also be allowed. They would be prohibited from receiving double treatment with yoga or mindfulness.

### 3.5. Participant Timeline

The participants’ timeline is displayed in Table 1.

## 4. Outcomes

Blind evaluators to randomization will assess all participants using the telephone-based survey before random assignment (Pre-test), the week following the last session of the intervention (post-test), and then after 3- and 6-months follow-up. It is noteworthy to mention that only the Montreal Cognitive Assessment outcome would be measured via live video call at baseline and post-intervention, 3- and 6-months follow-up.

### 4.1. Primary Outcomes

(1) Cognitive Function will be measured using a Spanish version of the Montreal Cognitive Assessment (MoCAS1-2) [62] is a short, easy-to-use, and useful test for diagnosing MCI and mild dementia. The test is valid in Chile, each of which has its own evaluation standard. The cut-off points for MCI and mild dementia are <21 and <20, respectively, with sensitivity and specificity rates of 75% and 82% for MCI and 90% and 86% for mild dementia.

### 4.2. Secondary Outcomes

(2) Physical function will be assessed using a:

(I) The Barthel Index (BI) [63] to measure performance in activities of daily living (ADL) [64]. Ten variables describe the capability of the person to carry out ten basic ADLs and mobility, a higher number reflecting a greater ability to function independently with mobility and self-care ADL.

(II) The Lawton Instrumental Activities of Daily Living Scale (IADL) [65] will be used to assess the independent living skills of participants. It measures functional ability as well as deteriorations and improvements over time. The protocols track eight functional domains through self-report, which measures everyday functional capability in the elderly. This would be performed by evaluating a more complex set of behaviors such as telephoning, shopping, food preparation, housekeeping, laundering, use of transportation, use of medicine, and financial behavior. Each area measured by the scale relies on either cognitive or physical function. Nonetheless, all require some degree of both [66].

(3) Psychological well-being will be assessed through the Pemberton happiness index (PHI) [67], which is a self-report questionnaire including two components of well-being. The first, with 11 items, assess the overall well-being and its four domains: general, psychological (eudaimonia), and subjective well-being (hedonia), as well as social welfare. The second, with 10 items, assess well-being experienced as a result of positive and negative experiences from the previous day. It is validated for the Spanish and Mexican populations. The internal consistency was 0.89 and the inter-item correlations varied between 0.31 and 0.56. The instrument has also been validated using samples from Chile, Cuba, and Uruguay, showing adequate psychometric properties [68].

(4) Geriatric Anxiety Inventory (GAI) [69] is a self-report measure designed for measuring general anxiety in older people. It consists of 20 items, measuring somatic, cognitive, and affective anxiety symptoms graded on a four-point scale of anxiety severity. Participants would be asked to rate anxiety symptoms by showing how often they have experienced each of the symptoms over the past week through a 4-point Likert scale ranging from 0 (not at all) to 3 (all time). Possible scores are in the range of 0 to 75, where high scores indicate high levels of anxiety [70]. It has been validated in Chile, showing a unidimensional factor structure with a high-reliability index of internal consistency with a = 0.931.

(5) The Geriatric Depression Scale (GDS-5) [71] is a depression screening measure that has been validated in a Chilean elderly population. It consists of 5-items questions for detecting geriatric depression. The five items are: (1) “Are you basically satisfied with your life?”, (2) “Do you often get bored?”, (3) “Do you often feel helpless?”, (4) “Do you prefer to stay at home rather than going out and doing new things?” and (5) “Do you feel pretty worthless the way you are now?”. A score of 2 or higher is indicative of possible depression. The GDS-5 demonstrated internal consistency of 0.73, construct reliability of 0.83, and one-dimensional structure [72].

## 5. Covariates

Information regarding Socio-demographic background, including sex, age, and education; diagnosed diseases (cardiovascular diseases) will be collected in addition to aforementioned outcomes through self-reported by the participants during the clinical interview in this study and controlled as a covariate in order to explore their potential modifying effects on the primary outcome (cognitive function) in MCI, as evidence reported aforementioned variables as a risk factor for progression from MCI to Alzheimer-type dementia [73,74,75].

### 5.1. Strategies to Promote Participant Retention and Complete Follow-Up

This clinical trial will not provide incentives to increase the retention of participants. Participants will be given telephone calls in case they have doubts regarding the intervention program and its contents. The neurologist in charge of evaluation for the diagnosis of patients with MCI will keep an up-to-date record of the participants’ contact information to prevent data loss during the follow-up period. Participants who decide to withdraw from the study will no longer complete follow-up assessments [76].

### 5.2. Data Management and Confidentiality

The data collected from each participant will be directly stored in electronic forms when participants directly enter the survey or by the recruiters or the outcome evaluators during the trial. After completion, these electronic forms will be submitted online through a safe platform to be automatically downloaded to a unique and encrypted database. No copies of the data will be kept on the electronic devices used in the study (e.g., smartphones, tablets, and personal computers) [76]. Data will be stored in a centralized database [76]. Tin order to protect the confidentiality of participants, other measures will be taken. Once data are registered, the identification of each patient will be eliminated from the database and will be replaced with an ID code. This will make it possible to work with anonymized data, thus protecting the participants’ confidentiality.

## 6. Analysis

Descriptive analyses of relevant variables will be conducted for the total sample and for each group separately in order to assess the balance between the intervention group and the control group after randomization, thus, for categorical variables, the percentage and frequency will be presented for each intervention group, and for continuous variables, means and standard deviations for each intervention group, along with the mean difference between the groups, 95% CI, and *p* value will be presented. In order to determine if there are significant differences in pre and post-evaluation between groups, a mixed design analysis of the variance model with repeated measures (ANOVA) will be performed. One factor, between-subject factor, would consider a “group” (composed of older MCI in the YBM intervention group and older MCI in the active control group with PSE), and the other factor, repeated measures or within subjects’ factor, would be considered as “time” (composed by “pre”, “post”, & “follow up” measures) for primary and secondary outcomes.

In order to report the strength of the YBM intervention, the effect size will be calculated as well, level of significance will be set at *p* < 0.05 with 95% CI, Therefore, based on the repeated measure Anova, effect size calculation would be performed using the standardized mean difference (SMD), Cohen’s d [77]. According to the main outcomes of the study and the condition of the intervention program, since educational level, age and sex are considered as potential confounders o covariates, so they will be controlled at baseline in the Ancova model, and it will enter to model as factor or covariance using the continuous measure. Hence, effect size calculation would be performed using Partial Eta^2^. Moreover, subgroup analyses will be performed to test the possible mediator as well. All the analyses will be based on the intention-to-treat principle, accounting for loss-to-follow-ups, and will be performed using Stata, version 17.0.

### 6.1. Plans to Give Access to the Full Protocol and Participant Level-Data

Access to the full protocol and participant-level data upon reasonable request to the corresponding author.

### 6.2. Patient and Public Involvement

Patients would be involved in the design, conduct, reporting, or dissemination plans of this research. Refer to the Methods section for additional details.

### 6.3. Dissemination Plans

The results of primary and secondary outcomes of this clinical trial will be available to the scientific community in a peer-reviewed journal devoted to the topic of interest.

### 6.4. Composition of the Data Monitoring Committee, Its Role, and Reporting Structure

As this trial involves minimal risks to the patients, no data monitoring committee will be established.

### 6.5. Adverse Event Reporting

The risks related to YBM intervention are minimal. Consequently, adverse events are not expected.

### 6.6. Strategies for Communicating Important Protocol Amendments

Any modifications to the trial protocol which may affect the safety of the participants must be approved by the Scientific Ethics Committee of the Clinical Hospital of the Universidad de Chile.

## 7. Discussion

With the aging of the global population aging, cognitive impairment (CI), including dementia and mild cognitive impairment (MCI), has become an important issue in public health [78,79]. The prodromal stage of dementia is also referred to as mild cognitive impairment (MCI) [80], which is characterized by a measurable decline of cognition without losing the capacity to perform the activities of daily life. In addition, MCI patients often manifest symptoms such as depression, anxiety, apathy, and irritability [81], which increases the likelihood of progressing to dementia [82]. Moreover, frailty is a multidimensional condition typical of elders, which are strongly associated with cognitive impairment [83]. Recent evidence revealed a significant correlation between physical and cognitive decline in the elderly with frailty [84]. Frail senior citizens have a higher risk of functional decline, hospitalization, and mortality [84].

Current pharmacological treatment options for MCI are limited [13], emphasizing the need for effective non-pharmacological treatments. Mindfulness-based interventions have shown a promising effect on several health factors related to the increased risk of progression from MCI to dementia, for instance, depression, stress, cognitive decline, as well as change in brain structure and immune system [32,51,56].

The present study would be the first randomized controlled trial aimed at evaluating the effectiveness of yoga-based mindfulness (YBM) intervention versus psycho-educational sessions in improving cognitive and physical functions and mental health outcomes for older adults with mild cognitive impairment (MCI) in Chile.

The intervention has been adopted. There is fresh evidence of the effectiveness of mind-body interventions, including yoga and mindfulness, in older adults with MCI. Currently, there is no local evidence of any psychosocial intervention based on mind-body therapy focused on older people with MCI. In Chile, adults over 60 will increase from the current 15.7% of the population to 32.9% by 2050 [5]. Therefore, aging is a highly relevant issue for the social and public health systems, especially in Chile, a country with great social and health inequalities [24].

It is important that Chile start collecting scientific evidence on the mental health of older people and on feasible and effective interventions for this population. In the case of cognitive impairment, the evidence is not robust and seems to reaffirm the need for early interventions. Present investigation can be an important contribution to the knowledge of older adults with MCI, who are at risk of conversion to AD. If the intervention proves to be effective, it would be of great interest and would enable its replication in a larger study, which would be relevant to national public health policy. The follow-up evaluations would enable researchers to evaluate its efficacy and to study its effects in greater depth. It is important to note that the aging process requires that society be able to fulfill the needs of the elderly Chilean people in terms of public policies, programs, and resources specifically designed for this population that contribute to active aging [85]. The results could help future researchers evaluate the potential of the intervention program to be part of an international strategy aimed at strengthening the cognitive and physical function as well as the mental health of older people with MCI in Latin America.

## 8. Conclusions

The YBM intervention protocol based on video recording has been adapted and designed. It is expected to be implemented as an acceptable and effective non-pharmacological option for older people with MCI. Providing evidence-based programs as a possible preventive treatment strategy for Alzheimer’s disease in the elderly has relevant implications for public mental health services in Chile.

## Figures and Tables

**Table 1:** Participants Timeline.

	Study Period						
	Enrolment	Allocation	Post-Allocation				Close-Out
Timepoint **	−t_1_	0	t_1_	t_2_	t_3_	t_4_	t_5_
Enrolment			Baseline	Intervention	Post	3M	6M
Eligibility screen	X						
Informed consent	X						
Allocation		X					
Intervention							
YBM Intervention				X			
Control							
PES				X			
Assessments							
MoCA	X				X	X	X
Demographic	X						
BI			X		X	X	X
IADL			X		X	X	X
PHI			X		X	X	X
GAI			X		X	X	X
GDS-5			X		X	X	X

Time points are: t_1_ baseline, t_2_ intervention period, t_3_ post-line evaluation, t_4_ 3 months follow up, t^5^ 6 months follow up; ** Montreal Cognitive Assessment (MoCA), Barthel Index (BI), Lawton Instrumental Activities of Daily Living Scale (IADL), Pemberton happiness index (PHI), Geriatric Anxiety Inventory (GAI), Geriatric Depression Scale (GDS-5).

## Data Availability

The datasets used and/or analyzed during the current study will be available from the corresponding author upon reasonable request.

## Abbreviations

MCI: Mild cognitive impairment; CI: Cognitive Impairment; AD: Alzheimer’s; MoCA: Montreal Cognitive Assessment; FDA: Food and Drug Administration; MET: Memory Enhancement Training; YBM: Yoga based- mindfulness; MBI: Mindfulness-based Intervention; MBSR: Mindfulness-based Stress Reduction; DMN: Default mode Network; PES: Psycho-educational Sessions; IADL: Instrumental Activities of Daily Living Scale; GAI: Geriatric Anxiety Inventory; GDS: Geriatric Depression Scale; BI: Barthel Index; MCID/MCIC: Minimal Clinically Important Difference or Change; PHI: Pemberton happiness index.
